# Central Serous Chorioretinopathy in a 14-year-old atopic boy: a case report

**DOI:** 10.1186/s13052-022-01386-4

**Published:** 2023-01-03

**Authors:** Stefano Ranno, Liviana Fontanel, Edoardo Ruggiero, Paolo Nucci, Paolo Radice, Simone Donati

**Affiliations:** 1 Ophthalmology Department, Circolo and Fondazione Macchi Hospital, ASST Sette Laghi, Varese, Italy; 2 University of Insubria, Department of Medicine and Surgery, Via Guicciardini 9, 21100 Varese, Italy; 3 Eye Vision Ophthalmology Center, Como, Italy; 4 Department of Clinical Sciences and Community Health, University of Milan, Milan, Italy

**Keywords:** Central serous Chorioretinopathy case report, Corticosteroid adverse effects, Corticosteroids, Pediatric ophthalmology

## Abstract

**Background:**

Corticosteroids are widely used in medicine. Few cases of central serous chorioretinopathy (CSC) have been reported following topical corticosteroid administration. We describe the first case of pediatric CSC related to topical corticosteroid administration.

**Case presentation:**

A 14-year-old boy presented with decreased vision, pigment epithelial detachments, and serous retinal detachments in the right eye after starting treatment for atopic dermatitis with Betamethasone Valerate 0.1% topical ointment. His condition resolved 2 weeks after discontinuing the steroid and administering Bromfenac 0.9 mg/ml eyedrops.

**Conclusions:**

Although the pathogenesis of CSC is poorly understood, ophthalmologists should be informed about the potential link between CSC and topical corticosteroid treatment, and they should be aware that CSC might, albeit infrequently, affect children.

## Background

Central serous chorioretinopathy (CSC) is an ocular disease that causes an idiopathic serous detachment of the retina and a fluid leakage from the choroid through a defect in the retinal pigment epithelium (RPE) outer blood-retina barrier [1]. CSC is a common cause of visual impairment in the working-age population and has been estimated as the fourth most frequent non-surgical retinopathy after age-related macular degeneration, diabetic retinopathy, and retinal vein occlusion [2]. Patients often experience loss of central vision, central scotoma, micropsia or metamorphopsia. Visual acuity may only be moderately decreased, and there may be a hyperopic shift [2]. Although many cases resolve spontaneously with minimal sequelae, 30-45% of patients have recurrence and a poorer visual prognosis [3]. CSC typically affects men aged 25 to 50 years, but it may occur in women or older individuals [3]. Although the pathogenesis remains unknown, risk factors include type A personality, Cushing disease, pregnancy, and corticosteroid use [1]. Actually, there are only five reported pediatric CSC cases [4–8]. To the best of our knowledge, we report the first pediatric CSC case associated with topical corticosteroid use.

## Case presentation

A 14-year-old boy presented with a 2-week history of decreased visual acuity in the right eye.

The boy suffered from atopic dermatitis (AD) with facial and scalp involvement and had been treated by a dermatologist since he was 5 years old. The disease had been well controlled for several years by skin hydration with soaking baths and use of topical ointments.

However, skin lesions had worsened in the past 2 years and the patient was prescribed Betamethasone Valerate 0.1% topical ointment 3 times a day. Although the patient was instructed to stop the therapy after 5 weeks, he continued using the ointment 3 times a day for about 6 months, after noticing significant improvement.

He presented at our attention complaining decreased visual acuity in the right eye. On ocular examination, his best corrected visual acuity measured 20/25 in his right eye and 20/20 in his left eye. Intraocular pressure was normal and anterior segment examination in each eye was unremarkable. Posterior segment examination of the right eye showed a large serous retinal detachment involving the posterior pole. Posterior segment examination of the left eye was normal.

**Fig. 1:**
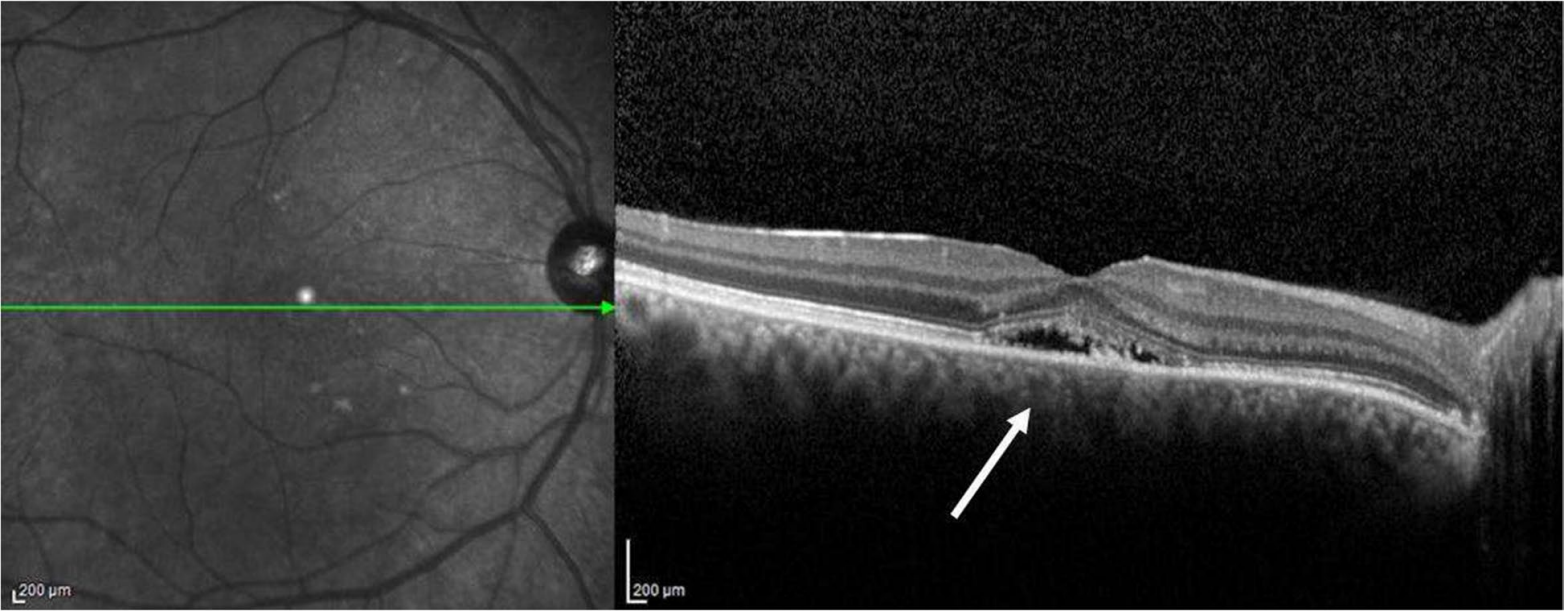
Right eye, Baseline. Infrared funduscopic image (left) and macular OCT B-scan (right). Presence of subretinal fluid causing a circumscribed area of serous retinal detachment in the macular region (white arrow), associated with elongated photoreceptor segments and irregular retinal pigment epithelium layer

Therefore, we decided to perform a macular optical coherence tomography (OCT) examination (Spectralis HRA; Heidelberg Engineering, Heidelberg, Germany). OCT examination in the right eye (Fig. 1) revealed the presence of a detachment of the neurosensory retina in macular region, associated with elongated photoreceptor segments and irregular RPE. Multiple associated pigment epithelial detachments were located inferonasally and inferotemporally to the fovea. In the left eye OCT examination showed normal finds.

Lastly, the blood pressure values were within normal ranges and the patient claimed to have a peaceful mind.

Structural retinal findings associated to clinical signs and history addressed the diagnosis to the CSC, probably linked to the persistent topical steroid treatment.

We decided to treat the patients with Bromfenac 0.9 mg/ml eye drops 3 times a day and to discontinue Betamethasone Valerate ointment. The therapy was well-tolerated by the patient and no side effects were reported.

**Fig. 2:**
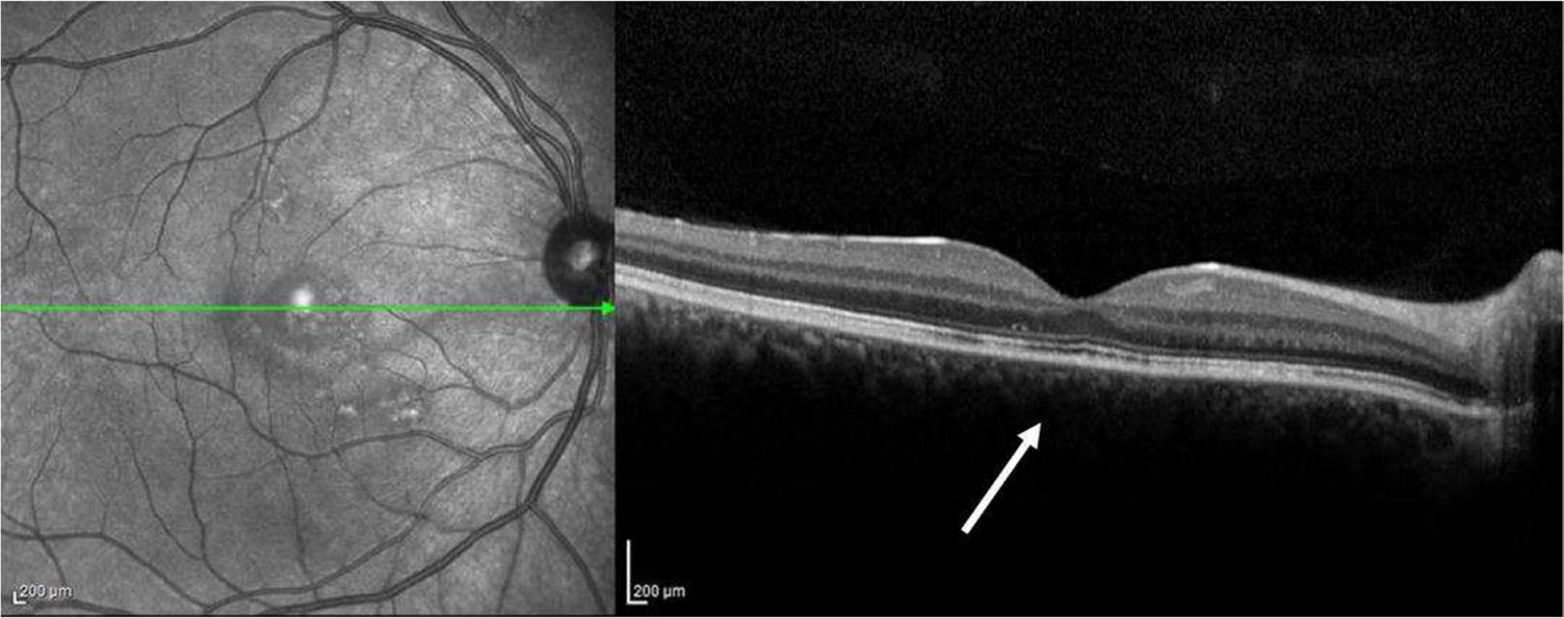
Right eye, Follow up at 2 weeks after stopping corticosteroid ointment. Infrared funduscopic image (left) and macular OCT B-scan (right). Complete remission of neuroepithelium detachment (white arrow), with resolution of subretinal fluid and photoreceptors - retinal pigment epithelium irregularity

At 2 weeks follow-up, OCT examination showed a complete resolution of neuroepithelium detachment (Fig. 2), associated to a restoration of normal visual acuity.

## Discussion and conclusions

This atypical case of CSC in a pediatric patient resolved promptly on cessation of the topical steroid and administration of Bromfenac eye drops.

The pathophysiology of CSC is poorly understood. Guyer and colleagues suggested that the pathogenesis of CSC may be choroidal vascular hyperpermeability. They noted diffuse hyperpermeability around active leakage sites seen with indocyanine green videoangiography (ICG-V) but not with fluorescein angiography (FA). Therefore, they concluded that hyperpermeability was at the level of the choroid rather than the RPE [9]. An alternative theory suggests that CSC results from dysfunction of the RPE which causes a reverse in fluid movement in a chorioretinal direction [10].

CSC induced by the systemic use of steroids was first reported in 1984 in two patients that developed serous macular detachment upon initiation of systemic Betamethasone therapy for retrobulbar neuritis [11]. However, the best evidence for an association between corticosteroid use and CSC comes from two large, retrospective case-control studies [12, 13]. Tittl and colleagues conducted the first of these, studying systemic factors associated with CSC [12]. This study included a total of 230 CSC cases and 230 age and sex-matched controls. They found that 21 CSC subjects were using corticosteroid medications, whereas 7 control subjects were using corticosteroids. This difference yielded an odds ratio of 3.2 (95% CI 1.3 to 7.70, *P* = 0.0063). Carvalho-Recchia et al. published the first report of a consecutive series of patients with acute CSC studied prospectively for an association with corticosteroids [13]. They found a statistically significant difference in corticosteroid exposure between study patients and controls.

The mainstay of Atopic Dermatitis treatment is represented by topical corticosteroids, while several routes of steroid administration have been discussed in the literature. These medications reduce inflammation and pruritus primarily by inhibiting the transcriptional activity of various proinflammatory genes. Topical corticosteroids are available in a wide range of potencies, from the least potent Group 1 preparations (e.g. Hydrocortisone 1% ointment), to the most potent Group 7 preparations (e.g. Clobetasol Propionate 0.05% ointment). The greater the potency of topical corticosteroid used, the greater the risk of systemic and local side effects [14]. Betamethasone Valerate 0.1% is in group 3 (high potency topical corticosteroids).

Despite that, it was suggested that steroid-induced CSC may be related to an idiosyncratic response in selected vulnerable individuals rather than to a dose-dependent effect, since very low doses can induce CSC episodes [15].

CSC has been associated with topical steroid use in several case reports. In 2004, Karadimas and colleagues reported two cases of presumed topical steroid-associated CSC [16]. Fernandez and colleagues reported a suspected case of topical steroid-associated CSC, in which a 43-year-old female developed CSC after 1 month of topical steroid use for lichen planus [17]. Ezra and colleagues also reported a case of a 25-year-old male with psoriasis and 15 years of topical steroid use. He experienced a single episode of CSC with resolution upon steroid cessation [18].

While CSC has previously been linked to systemic corticosteroid use, it is rarely linked to topical administration.

Idiopathic CSC in children has been reported in a few case reports [5–8], however this condition has never been linked to topical corticosteroid therapy in young patients.

In light of this, we assume that this may be the first pediatric case of CSC related to transdermal steroid treatment.

Poor imaging is a limitation of this case report, because CSC diagnosis is typically based on a serous retinal detachment described on OCT examination and confirmed by FA, which reveals early-phase localized dye leakage and late-phase dye pooling under the sensory retina. However, we chose not to perform FA on our young patient because OCT scans clearly revealed the diagnosis.

In conclusion, it should be kept in mind that CSC can affect children as well as adults, and this case outlines the importance for ophthalmologists to carefully assess CSC patients for local and systemic corticosteroid use.

## Data Availability

The datasets used and analyzed during the current study are available from the corresponding author on reasonable request.

## Abbreviations

AD: Atopic dermatitis
CSC: Central serous chorioretinopathy
FA: Fluorescein angiography
ICG-V: Indocyanine green videoangiography
OCT: Optical coherence tomography
RPE: Retinal pigment epithelium
